# Consent and Deidentification of Patient Images in Dermatology Journals: Observational Study

**DOI:** 10.2196/37398

**Published:** 2022-07-06

**Authors:** Japbani K Nanda, Michael Armando Marchetti

**Affiliations:** Dermatology Service, Department of Medicine, Memorial Sloan Kettering Cancer Center, New York, NY, United States

**Keywords:** consent, deidentification, journal, images, publication, privacy, readability, internet, dermatology, photo, privacy, patient rights, publication, publish, permission, publication, data sharing

Publication of patient images contributes to research and education in dermatology. However, it is important to protect patients’ privacy and rights. The Committee on Publication Ethics (COPE) and the International Committee of Medical Journal Editors (ICMJE) have provided best practices and recommendations, respectively, for the protection of patients’ rights in scholarly publications [1,2]. Nonetheless, requirements for the deidentification of patient images and for the acquisition of consent to publish such images vary across governing bodies and journals. Our objective was to describe leading dermatology journals’ instructions regarding deidentification and consent to publish patient images as well as the content and readability of consent forms.

This study was exempt from institutional review board review as data were publicly available. Themes regarding the publication and deidentification of patient images, as well as the acquisition of consent, were extracted from COPE and ICMJE [1,2]. On June 9, 2021, the top 20 dermatology journals were determined using Google Scholar, which ranks journals based on the h5-index [3]. Guidelines, instructions for authors, submission checklists, and consent forms on the journals’ websites were reviewed for criteria embodied by the themes extracted from COPE and ICMJE. Legal clauses in consent forms were summarized. Available consent forms were prepared and then assessed for readability using Microsoft Word (Microsoft Corporation), which calculates the Flesch-Kincaid Grade Level (FKGL) [4]. FKGL considers average sentence length and the average number of syllables per word to provide a corresponding US grade level rating [4].

A total of 19 (95%) journals’ online instructions instructed authors to obtain written consent or permission for the publication of patient images (Table 1). The specific instances in which consent was required varied and included recognizable, identifying, identifiable, or possibly identifiable images; images that may, could be used to, could, or potentially identify the person; images in which the person could or can be identified, including by the patient; only if the patient’s face is completely identified; or any or all patient images, regardless of whether a patient is or is not identifiable. Some journals provided specific guidance on identifiable features, such as facial features (n=5), tattoos (n=1), and jewelry (n=1). A total of 11 consent forms were identified from 10 journals (Table 2). All forms emphasized that the individual in a published image may be identified or that anonymity cannot be guaranteed. The average FKGL was 15.3 (range 12.1–22.8).

Instructions regarding the deidentification of patient images and acquisition of consent for publication differed across dermatology journals and incorporated various elements from COPE and ICMJE [1,2]. Most leading dermatology journals instructed authors to obtain written consent or permission to publish patient images. This is in contrast to a study that found that approximately 52% of dental, oral surgery, and otorhinolaryngology journals had a policy regarding clinical images [5]. Although readability scores should be used with caution, consent forms were difficult to read and were written, on average, at a college level based on an FKGL score of 15.3. It has been recommended that materials for patients should be written at the sixth-grade level or lower [6]. Consideration should be given to enhancing consent form readability, which may improve patient understanding. Although we analyzed a small subset of journals from a specific subspecialty, our findings may raise awareness of the need to protect patients’ right to confidentiality by implementing consistent policies for the publication of clinical images.

## Figures and Tables

**Table 1.** Instructions for authors regarding deidentification, publication, and consent for patient images.

Criteria	Frequency among top 20 dermatology journals^a^, n (%)
Statement on requirement for consent or permission regarding patient images	19 (95)
Written or signed consent or permission required	18 (90)
Patient or patient representative to be informed that published content may be available on the internet	4 (20)
Patient or patient representative to be shown the manuscript that will be published	4 (20)
Publication of identifying information only if it is essential for scientific or scholarly purposes	11 (55)
Black bars or masking of the eyes or face are inadequate or not recommended	9 (45)
Recommend eye bar, black bar, or masking to anonymize	1 (5)
Recommend cropping of images or cropping performed by journal for deidentification	4 (20)

a Top 20 dermatology journals per Google Scholar h5-index, where h is the largest number of published articles with at least h citations for each article [3] (listed in alphabetical order): *Acta Dermato-Venereologica*; *American Journal of Clinical Dermatology*; *Anais Brasileiros de Dermatologia*; *British Journal of Dermatology*; *Clinics in Dermatology*; *Contact Dermatitis*; *Dermatologic Clinics*; *Clinical, Cosmetic and Investigational Dermatology*; *Dermatologic Surgery*; *Experimental Dermatology*; *Indian Journal of Dermatology*; *International Journal of Dermatology*; *JAMA Dermatology*; *Journal of Dermatological Science*; *Journal of Dermatological Treatment*; *Journal of Investigative Dermatology*; *Journal of the German Society of Dermatology*; *Journal of the American Academy of Dermatology*; *Journal of the European Academy of Dermatology and Venereology*; and *The Journal of Dermatology*.

**Table 2.** Characteristics of patient consent forms for the publication of images.

Criteria	Frequency among consent forms^a^, n (%)
Patient or signer to be shown the manuscript that will be published, or patient or signer may waive this opportunity	6 (55)
Patient or signer informed that published content may be available on the internet	7 (64)
Consents to publication of case information or photograph	11 (100)
Understands they may be identified or indicates that anonymity cannot be guaranteed	11 (100)
Name of patient or name of person signing	11 (100)
Name of person who explained the form, author, or doctor	10 (91)
Contact information of person who explained the form, author, or doctor	2 (18)
Indicates that signing does not remove the right to privacy	2 (18)
Indicates that the patient or signer has the right to revoke consent, but after publication, revocation of consent is not possible	4 (36)
Statement on financial benefit or lack thereof	2 (18)
Release to Affiliates, Subsidiaries, Third Parties or Other Websites	9 (82)
Release of Claims Clause	3 (27)
Choice of Law Clause	2 (18)
In Perpetuity Clause	2 (18)
Defamation Clause	1 (9)
Attorney’s Fees Clause	1 (9)

a A total of 11 consent forms were provided online by the following 10 dermatology journals (listed in alphabetical order): *Acta Dermato-Venereologica*; *American Journal of Clinical Dermatology*; *Anais Brasileiros de Dermatologia*; *British Journal of Dermatology*; *Clinical, Cosmetic and Investigational Dermatology*; *Contact Dermatitis*; *Experimental Dermatology*; *JAMA Dermatology*; *Journal of Dermatological Treatment*; and *Journal of the American Academy of Dermatology*.

## Abbreviations

COPE: Committee on Publication Ethics
FKGL: Flesch-Kincaid Grade Level
ICMJE: International Committee of Medical Journal Editors
